# Realising the ambivalent nature of H_2_O_2_ in oxidation catalysis – its dual role as an oxidant and a substrate

**DOI:** 10.1039/d5dt01106j

**Published:** 2025-06-11

**Authors:** C. Maurits de Roo, Marika Di Berto Mancini, Wesley R. Browne

**Affiliations:** a Faculty of Science and Engineering, University of Groningen Nijenborgh 3 9747AG Groningen The Netherlands w.r.browne@rug.nl

## Abstract

H_2_O_2_ is a desirable terminal oxidant due to its good atom economy with H_2_O being the only by-product when used productively. Its relative stability is advantageous in transport and storage, meaning that catalysts can both activate and direct its oxidising power towards selective oxidation of organic substrates. Wasteful disproportionation of H_2_O_2_ (into H_2_O and O_2_) is a well-recognised challenge and receives little, if any, attention in catalyst design. Nevertheless, understanding how H_2_O_2_ reacts during catalysed oxidations is essential to avoid inefficient use of H_2_O_2_, and, more importantly, hazardous conditions in which large amounts of O_2_ are released by disproportionation. Reaction progress monitoring is an essential component in catalyst development, typically focusing on substrate conversion/product yield. In this frontier article, we advocate for multi-spectroscopic reaction progress monitoring in which all reaction components, including the oxidant and O_2_, are tracked over the course of catalysed reactions to establish comprehensive time resolved mass balances. This approach provides insight into the reaction pathways that lead to disproportionation and the species responsible for it. We discuss selected cases to highlight the range of pathways possible and how these impact efforts towards reaction optimisation through catalyst design. In particular, the paradigm that the catalyst responsible for substrate oxidation is a distinct species from that responsible for H_2_O_2_ disproportionation, *e.g.*, catalyst degradation products, is likely often incorrect. Rather, various pathways are possible, *e.g.*, the same catalyst intermediate engages in both H_2_O_2_ and substrate oxidation. Various reaction pathways with respect to H_2_O_2_ consumption are discussed in the case studies. Our conclusion is that it is useful to consider that H_2_O_2_, in addition to being an oxidant, can compete with the intended organic substrate. This aspect is particularly important in efforts to elucidate reaction mechanisms and when redesigning catalysts rationally to improve performance, especially for use on large reaction scales where safety is paramount.

## Abstract 2

Activation of catalysts by H_2_O_2_ generates the intermediate species that react with the desired target organic functional group. The oxidation of H_2_O_2_ itself is often overlooked despite it being central to efforts to optimise catalysts by design.

## Graphical abstract

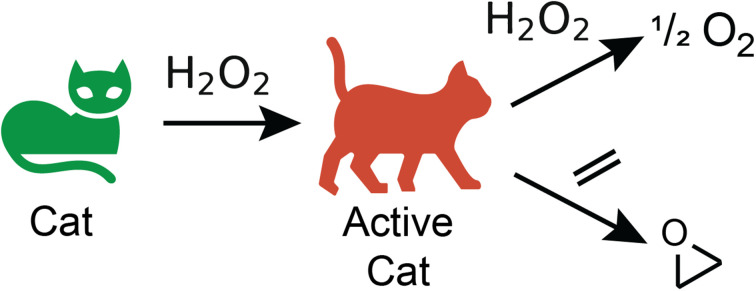

## Introduction

Despite that H_2_O_2_ is a potent oxidant, it is remarkably stable from a kinetic perspective and can be transported and stored relatively safely even at high concentrations. Its presence in biological systems is, however, invariably destructive and as a consequence nature has evolved many effective ways to ‘deactivate’ it safely, using oxidases and, when necessary, a wide range of catalases.^1,2^ Disproportionation (catalase) neutralises H_2_O_2_ by converting it to H_2_O and O_2_, but in doing so chemical potential is wasted, and worse, it can lead to other reactive oxygen species such as hydroxyl radicals, singlet oxygen, and the superoxide radical anion. Hence, the more effective way to deactivate H_2_O_2_ is to use it as a terminal oxidant releasing water and oxidising an organic substrate. In this way, the generation of reactive oxygen species is minimised.

In synthetic chemistry, this is the route of choice, using H_2_O_2_ as a reagent and H_2_O_2_ stands just behind molecular oxygen in terms of atom efficiency, with water as the only by-product in C–H hydroxylation, sulfoxidation and epoxidation, and complete atom economy in alkene dihydroxylation.^3^ These reactions are important in nearly all branches of the chemical industry, from polymer synthesis to medicinal chemistry.^4–7^

Nature inspires synthetic chemists in the design of ligands for transition metal catalysts to use H_2_O_2_ effectively, and over the last half-century, a small group of ligand families have emerged, primarily for oxidation catalysis based on iron and manganese, but also on other metals such as copper and cobalt.^8–13^ In these efforts, the focus is on selectivity, well-defined oxidative transformations, and to a lesser extent on efficiency, primarily in the productive use of H_2_O_2_. That is, while effectiveness in terms of substrate conversion is readily apparent, and indeed expected, in most studies in the literature, the fate of H_2_O_2_ in these reactions is unclear, more often than not, in large part due to the challenge in determining H_2_O_2_ consumed and O_2_ released during and after reactions. Moreover, when scaling a catalysed reaction up to an industrial process, with H_2_O_2_ as a terminal oxidant, efficiency in H_2_O_2_ use is critically important beyond activity and selectivity, as high concentrations of O_2_ can create hazardous conditions.

In this frontier article, we focus on the pathways involved in the competition between the oxidation of organic substrates and the oxidation of H_2_O_2_. The approaches needed to suppress the latter reaction depend on the mechanisms involved and hence knowledge of the nature of the species responsible is essential.

Reaction progress monitoring, in terms of the oxidation of organic substrates, is well developed, using in-line (Raman, FTIR, and NMR spectroscopy), at-line (GC and HPLC), and off-line (GC, HPLC, and NMR spectroscopy) techniques. In contrast, monitoring the concentration of H_2_O_2_ and O_2_ during a reaction is more challenging and we will discuss first the (spectroscopic) methods currently available.

## Analytical tools for reaction monitoring

**Fig. 1:**
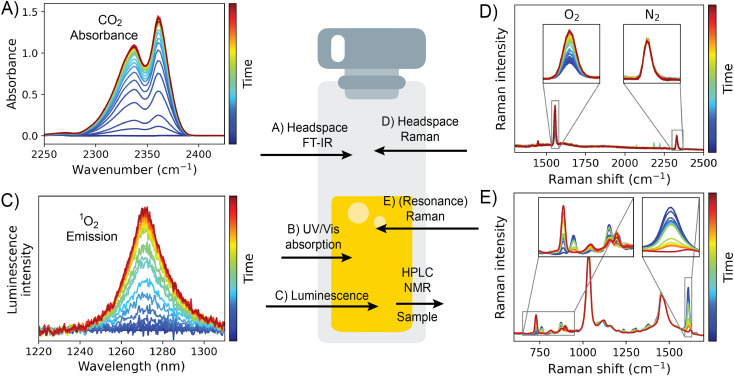
Analytical tools for reaction monitoring discussed here. (A) Headspace FTIR spectroscopy (CO_2_); (B) UV/vis absorption spectroscopy (*e.g.*, catalyst concentration); (C) luminescence spectroscopy (*e.g.*, ^1^O_2_ luminescence); (D) headspace Raman spectroscopy (gases that evolve from the reaction mixture); and (E) Raman (or resonance Raman) spectroscopy (reaction components in solution).

Spectroscopic tools that are readily available for monitoring the progress of reactions can be divided into those that monitor the liquid (reaction mixture) phase and those that monitor the headspace above the reaction mixture inside a reaction vessel (*e.g.*, a flask, a cuvette, *etc*.), or pipe in the case of flow chemistry (Fig. 1).

### Monitoring reactions in solution

Optical techniques, *e.g.*, UV/vis absorption spectroscopy, are invaluable in studying reaction mixtures when one or more of the reaction components, typically transition metal catalysts, show significant absorption (Fig. 1B). A 0.01–10 mM concentration window of the absorbing reaction components is typically required, dependent on the molar absorptivity of the compounds present, the optical path length, and the dynamic range of the spectrometer. The concentration of species of interest can be determined readily by the Beer–Lambert relation. Similarly, the liquid phase can also be monitored by (resonance) Raman and luminescence spectroscopy. Raman spectroscopy, although using visible/near-infrared light, provides vibrational spectra of the components at concentrations typically >50 mM or at (sub) mM concentrations where the wavelength of the laser used is resonant with an absorption band of a compound of interest (resonance Raman spectroscopy, Fig. 1E).^14^ Since the intensity of Raman scattering is linearly proportional to concentration, it is especially convenient for quantitative studies. Although less obvious in regard to reaction monitoring, luminescence spectroscopy is useful where the substrate, intermediate, product or other component present in the reaction mixture shows photo- or chemiluminescence (Fig. 1C).

As a case in point, the generation of singlet oxygen (^1^O_2_), *e.g.*, upon the reaction of MoO_4_ with H_2_O_2_, can be followed by the NIR emission of ^1^O_2_ (1269 nm).^15,16^ Quantification of ^1^O_2_ emission produced through chemiluminescence requires calibration of the spectrometer used to record luminescence intensities to relate it to the transient concentration of ^1^O_2_. The limit of detection and quantification of ^1^O_2_ emission for a particular spectrometer can be determined using the reaction of MoO_4_ with H_2_O_2_. ^1^O_2_ emission intensity is correlated also with the release of ^3^O_2_ into the headspace of a sealed cuvette, *e.g.*, by headspace Raman spectroscopy (*vide infra*).^17^

The reaction intermediates can be monitored by many other analytical techniques, not least online mass spectrometry as discussed in detail elsewhere.^18^ In addition to these time-resolved methods, sampling the liquid phase by withdrawing aliquots from the reaction mixture at certain time intervals for off-line analysis by NMR spectroscopy or chromatography methods (*e.g.*, HPLC, GC) is quite standard. However, care should be taken to ensure rapid quenching of the reaction in the sample to maintain time resolution. Furthermore, where reactions are carried out at low temperatures, heating the withdrawn sample before quenching can reduce the reliability of the measurement. It is in these aspects that in-line measurements are particularly beneficial. Finally, often for transition metal based oxidation catalysts, (spectro)electrochemical oxidation/reduction can be useful in identifying potential reaction intermediates,^19,20^ as, for example, in the study of [Mn(OTf)_2_(^R^PDP)] discussed below.^21^

### Monitoring the headspace over reaction mixtures

The headspace above the reaction mixture can be monitored for the production or consumption of gases by FT-IR spectroscopy (CO_2_) and/or headspace Raman spectroscopy (O_2_, N_2_, H_2_, Fig. 1D). Headspace FT-IR spectroscopy can be performed readily in ordinary glass reaction vessels as the spectral cut-off for glass is *ca*. 2000 cm^−1^, at a lower wavenumber than many gases of interest. Indeed, a simple approach is to lower a (sealed) cuvette so that the IR light travels through the headspace of the cuvette rather than the liquid phase (Fig. 1A). In this way the evolution of CO_2_ gas can be monitored and quantified using the pathlength and its molar absorptivity.^22^ Although Raman scattering from gases is much weaker than in the condensed phase, due to both differences in polarisability and more critically the number density in the confocal volume, headspace Raman spectroscopy is effective in monitoring the headspace above reactions for gases such as O_2_ (at 1556 cm^−1^), N_2_ (at 2332 cm^−1^), and H_2_ (at 4197 cm^−1^)^23^ (Fig. 1D).^24^ The gas of interest (usually O_2_ in the case of oxidation catalysis) may be quantified by relating the integrated Raman intensity of the *ν*_O−O,str_ band to the number of moles of the gas, following the ideal gas law and Henry's law,^17^ which accounts for the shift in equilibrium between the gas in the headspace and the gas dissolved in the liquid phase (due to the production/consumption of the gas in the closed volume).

In the next sections, the power of a multi-spectroscopic approach is exemplified by three case studies exhibiting three distinct types of interaction between a Fe- or Mn-catalyst and H_2_O_2_ with respect to substrate oxidation and H_2_O_2_ disproportionation.

## Case studies in elucidating reaction pathways

In the following examples, we show how understanding which species are responsible for catalysing disproportionation of H_2_O_2_ enables rational redesign of catalyst systems to minimise waste.

### Competition between H_2_O_2_ and organic compounds as substrates for the activated form of an oxidation catalyst

The [Mn(OTf)_2_(^R^PDP)] family of complexes (where R = H, OMe and ^R^PDP = *N*,*N*′-bis(2′′-(4′′-R-pyridylmethyl)-2,2′-bipyrrolidine)) and Mn(ii)/pyridine-2-carboxylic acid are two examples of catalysts in which the same reactive species is responsible for both substrate oxidation and H_2_O_2_ decomposition.

#### Oxidations with Mn(PDP) catalysts

**Fig. 2:**
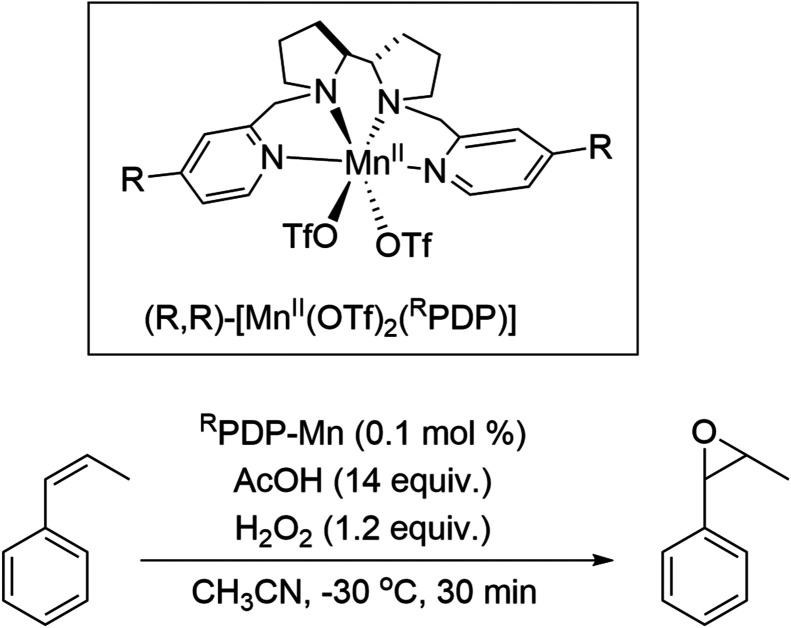
Manganese ((*R*,*R*)-[Mn(OTf)_2_ (^R^PDP)]) catalysed epoxidation of *cis*-β-methylstyrene with H_2_O_2_.^26^

The [Mn(OTf)_2_(^R^PDP)] family of complexes (where R = H in ^H^PDP–Mn and R = OMe in ^OMe^PDP–Mn) are remarkably effective in the (enantioselective) oxidation of organic compounds (C–H oxidation and epoxidation/dihydroxylation, Fig. 2).^25–27^

Oxidations with these catalysts are typically carried out with a small (20%) excess of H_2_O_2_ with respect to the organic substrate.^28^ Substituents on the pyridine rings show a considerable influence over the efficiency of the reactions, *i.e.*, conversion and product yields (Table 1). Indeed, although the effect of substituents on conversion is considerable, the effect on enantiomeric excess is all the more remarkable considering the lack of proximity of the substituent to the reaction centre. Since the substituents are in the *para*-position, steric effects are less pronounced than in the other positions, and hence the effect of the substituents on electron density at the metal centre is focused on in regard to rationalisation of trends observed.^26,29^

**Table 1:** Conversion and enantiomeric excess (ee) in the Mn-catalysed epoxidation of *cis*-β-methylstyrene^26^

Catalyst	R	Conversion (yield, %)	ee (%)
^H^PDP–Mn	–H	61 (38)	43
^OMe^PDP–Mn	–OMe	80 (59)	69
^Me^PDP–Mn	–Me	100 (67)	63
^Cl^PDP–Mn	–Cl	57 (33)	40
^Me_2_N^PDP–Mn	–NMe_2_	100 (75)	82
^CO_2_Et^PDP–Mn	–CO_2_Et	44 (22)	43

The difference in the performance of the variously substituted catalysts, *e.g.*, between ^H^PDP–Mn and ^OMe^PDP–Mn analogues (Table 1), and in particular the turnover number (TON), has been ascribed tentatively to the resilience of the catalyst toward inactivation/degradation, or to the maximum turn over frequencies (TOF) each catalyst can achieve and hence compete with unproductive reactions (disproportionation of H_2_O_2_).

A combination of resonance Raman, EPR, and UV/vis absorption spectroscopy under reaction conditions revealed the appearance of multi-nuclear Mn(iii) and Mn(iv) complexes, which are reminiscent of dinuclear manganese complexes that show activity in the disproportionation of H_2_O_2_ (*vide infra*).^21,30^ However, these species were shown, using in-line time resolved spectroscopy, to be mostly resting states. In line reaction monitoring of substrate conversion and evolution of O_2_ revealed that the differences in the efficiency of the ^OMe^PDP–Mn complex and its ^H^PDP–Mn analogue were in fact due to the effect substituents on the relative rates of substrate oxidation and H_2_O_2_ oxidation.^21^

**Fig. 3:**
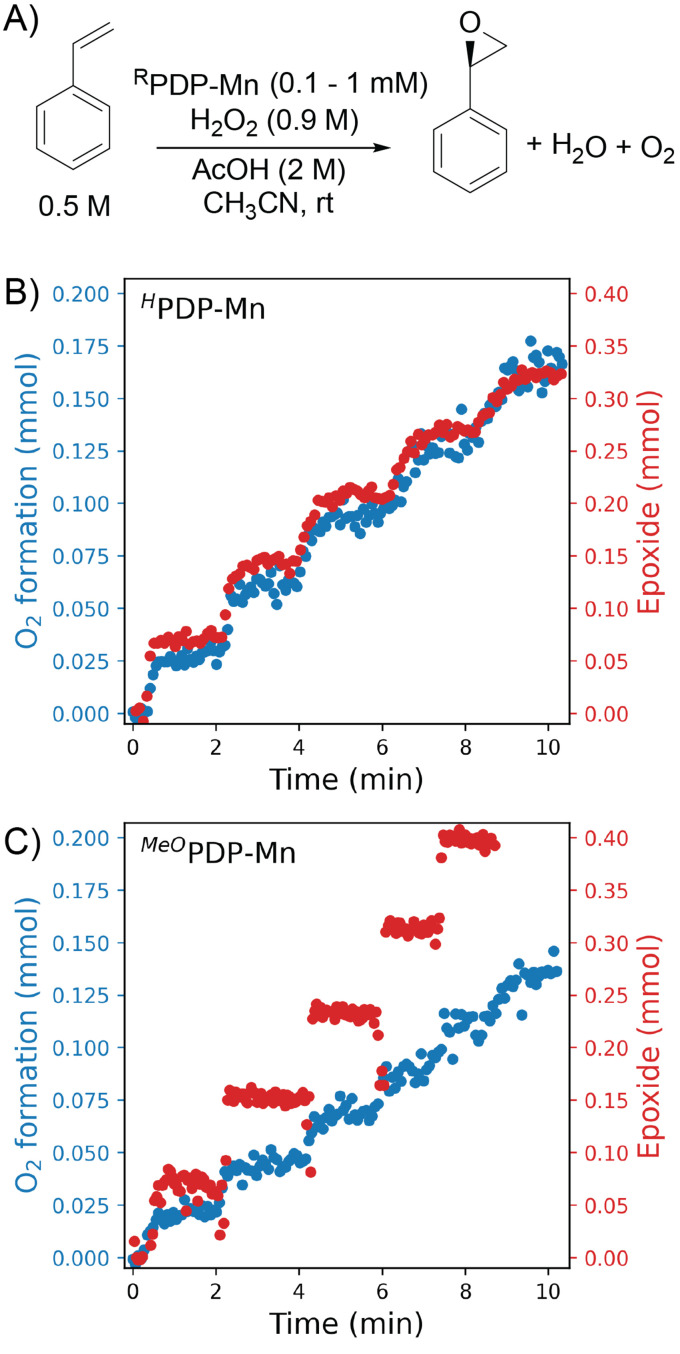
(A) Manganese catalysed epoxidation of styrene using [Mn(OTf)_2_(^R^PDP)]. For (B) [Mn^II^(OTf)_2_(^H^PDP)] and (C) [Mn^II^(OTf)_2_(^OMe^PDP)] the amount of O_2_ released (blue) into the headspace and epoxide formed (red) in the liquid phase, both determined by Raman spectroscopy. Reproduced from Kasper *et al*. (ACS 2023).^21^

The comparative study focused on the relative efficiency of the two catalysts in the epoxidation of styrene and disproportionation of H_2_O_2_ over the whole course of the reaction.^21^ Simultaneous headspace/liquid phase in line Raman spectroscopy was used to quantify the loss of H_2_O_2_/release of O_2_, as well as the formation of styrene oxide (Fig. 3). In these experiments, H_2_O_2_ was added dropwise over 10 min or longer. The spectroscopic data revealed that O_2_ was formed concurrent with alkene oxidation and that the relative efficiencies for the two reactions were constant over the entire period of H_2_O_2_ addition for both catalysts (Fig. 3).

However, the ratio of alkene oxidation to H_2_O_2_ is different for the two complexes. [Mn(OTf)_2_(^OMe^PDP)] favours alkene oxidation over H_2_O_2_ oxidation to a greater extent than [Mn(OTf)_2_(^H^PDP)]. These observations provided a strong indication that H_2_O_2_ and styrene were competitive substrates for the H_2_O_2_ activated catalyst, rather than that different species were responsible for alkene oxidation and H_2_O_2_ disproportionation. Furthermore, the data indicate that the substituents determine the overall efficiency of the catalysts by affecting the selectivity towards oxidation of alkene and H_2_O_2_.

The influence of substituents on the activity of the catalysts can be estimated from the turnover frequency (TOF) for the formation of epoxide over time after each drop of H_2_O_2_. The conclusion reached on this basis is that [Mn^II^(OTf)_2_(^OMe^PDP)] is a more active catalyst than [Mn^II^(OTf)_2_(^H^PDP)]. However, when the oxidation of H_2_O_2_ is taken into account, as well, the overall activity of the two catalysts is in fact similar. While it is uncertain whether the same species is responsible for both H_2_O_2_ and substrate oxidation, the constant relative reactivity over the entire course of the reaction suggests it is.^21^ In conclusion, this example showcases H_2_O_2_ as a competing substrate for the activated catalyst and how simultaneous in-line monitoring can reveal this.

#### Oxidations with Mn(ii)/pyridine-2-carboxylic acid

**Fig. 4:**
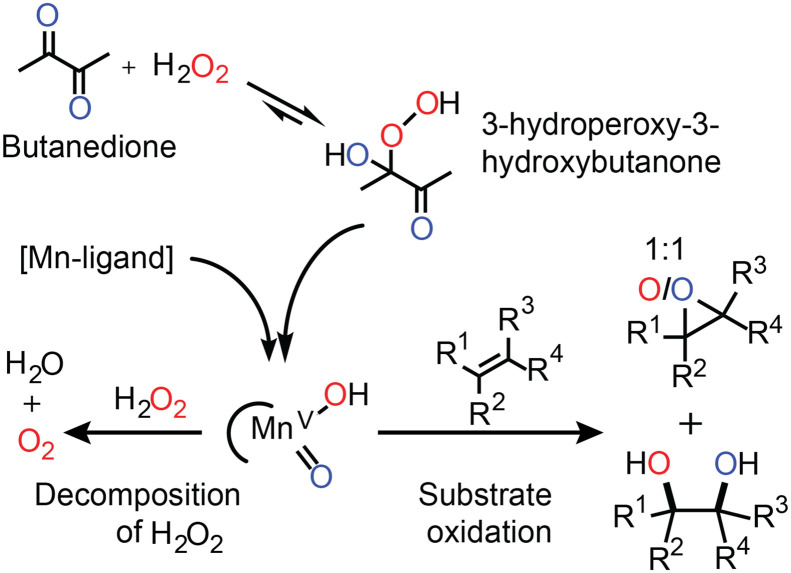
The activation of H_2_O_2_ by the *in situ* prepared Mn(ii)/PCA catalyst relies on the initial formation of a ketone-hydroperoxide adduct (*e.g.*, with butanedione). Organic substrates compete with H_2_O_2_ for the reaction with the activated manganese species (tentatively assigned LMn(v)O(OH)).^33^ The results of oxygen atom tracking with oxygen in H_2_O_2_ (red) and oxygen in butanedione (blue) are shown.^31^

That a catalyst shows competition between the desired oxidation of organic substrates and wasteful oxidation of H_2_O_2_ is not necessarily obvious. In particular, where good to excellent conversions are achieved for many substrates, H_2_O_2_ oxidation can be overlooked. A case in point is that of the oxidation catalyst prepared *in situ* from Mn(ii)/pyridine-2-carboxylate (PCA) and a ketone (Fig. 4).^31–33^

Elucidation of the mechanism by which this catalyst operates is challenging since the low concentration of Mn(ii) used means that direct observation of catalyst species is impractical. Nevertheless, in line spectroscopy is invaluable in monitoring changes to the major (>50 mM) components of the reaction mixtures and has allowed for a relatively detailed understanding of the role of each reaction component to be established (Fig. 4).^31–33^ For example, a ketone such as butanedione acts as a co-catalyst by forming the hydroperoxy-adduct with H_2_O_2_ from which the oxidising manganese species is formed (Fig. 4). The formation of this species is rate limiting, and unusually the reaction rate shows a zero-order dependence on the substrate concentration. However, the catalyst is still highly selective. For example, while in separate reactions, styrene and 1-phenyl-ethanol are oxidised with the same observed rate, in a mixture styrene is oxidised first with 1-phenyl-ethanol oxidation beginning only after most of the styrene had been consumed.

Although the catalyst is efficient with high to full conversion with many substrates, a small excess of H_2_O_2_ is still required to reach full conversion of alkene. The inefficiency can be ascribed to the concomitant oxidation of other reaction components (*e.g.*, the ketone used as a co-catalyst). However, headspace reaction monitoring together with ^18^O labelling (Fig. 4) confirmed that H_2_O_2_ oxidation also occurs.

**Fig. 5:**
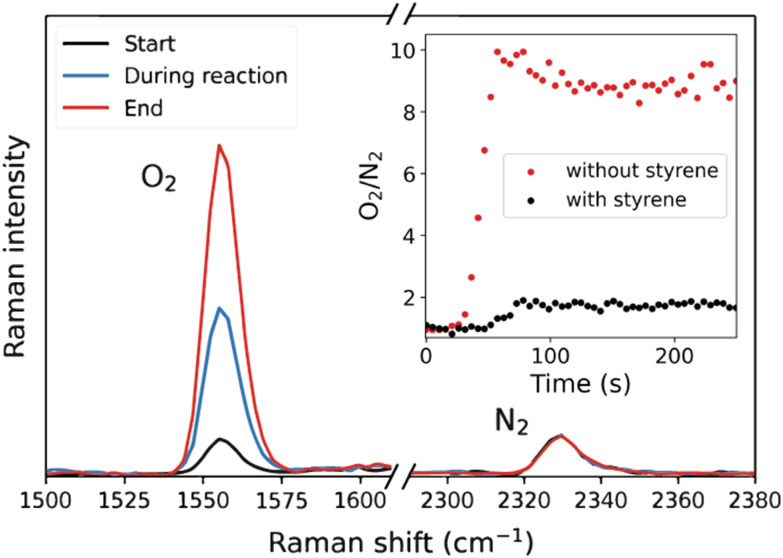
The release of O_2_ in the reaction of H_2_O_2_ catalysed by Mn(ii)/PCA was monitored by headspace Raman spectroscopy. In the presence of styrene, O_2_ is formed only after almost all styrene has been oxidised to styrene oxide (black dots), while in the absence of styrene O_2_ is released quantitatively (red dots) due to oxidation of H_2_O_2_.^31^

Monitoring the extent of O_2_ evolution during the oxidation of styrene indicates that disproportionation is only significant at the end of the reaction and hence contrasts with the observations made with the Mn-PDP catalysts (*vide supra*). Namely, as the Mn(ii)/PCA catalyst shows a zero-order dependence on the substrate, the evolution of O_2_ is expected only at the end of the oxidation of styrene. The catalyst shows preference for styrene as the substrate and only when it is consumed does H_2_O_2_ become a competitive substrate. Indeed in the absence of styrene, O_2_ evolves over the entire course of the reaction at the same rate at which styrene had been oxidised (Fig. 5).

Despite differences in the evolution of O_2_ over time between the Mn(ii)/PCA and Mn–PDP catalyst systems, the origin of wasteful H_2_O_2_ disproportionation is in both cases due to competition between the organic substrate and H_2_O_2_ for the oxidising manganese species. Hence, the overall catalyst efficiency depends on the competition for the activated catalyst.

### Identifying competing pathways in organic substrate oxidation and H_2_O_2_ decomposition through reaction monitoring and kinetic modelling

**Fig. 6:**
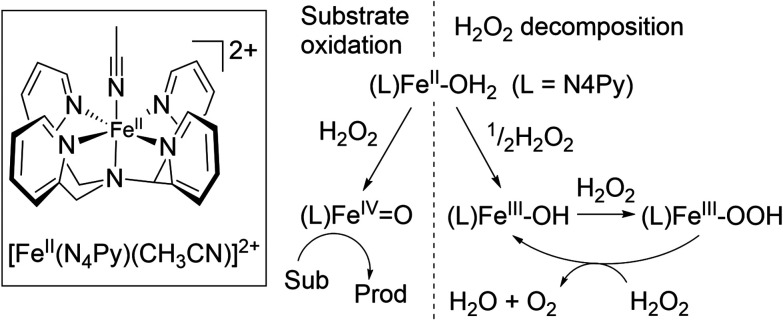
Proposed mechanism for [(N_4_Py)Fe^II^ (CH_3_CN)](OTf)_2_ with H_2_O_2_ in methanol.^17^

In contrast to the previous examples, the complex [(N_4_Py)Fe(ii)(CH_3_CN)]^2+^, where N4Py is (1,1-bis(pyridin-2-yl)-*N*,*N*′-bis(pyridin-2-ylmethyl)methanamine) (Fig. 6) is an example of an oxidation catalyst in which different species are responsible for substrate oxidation and/or H_2_O_2_ decomposition.^34–37^

[(N_4_Py)Fe(ii)(CH_3_CN)]^2+^ reacts with H_2_O_2_ to form an Fe(iv)=O species ([(N_4_Py)Fe(iv)=O]^2+^) that can oxidise organic substrates selectively.^38^ However, the complex reaches the Fe(iii) state relatively rapidly both through Fe(ii)/Fe(iv) comproportionation and by reduction of the initially formed [(N_4_Py)Fe(iv)=O]^2+^ through hydrogen atom transfer with H_2_O_2_, producing [(N_4_Py)Fe(iii)(OH)]^2+^ and the superoxide anion radical.^17,39^ The Fe(iii) complex, *e.g.*, [(N_4_Py)Fe(iii)(OCH_3_)]^2+^, also undergoes ligand exchange with H_2_O_2_ to form a relatively stable Fe(iii)–OOH species. Although this species can be viewed as the precursor to [(N_4_Py)Fe(iv)=O]^2+^*via* homolytic O–O bond cleavage, this reaction is remarkably slow.

Instead, in protic solvents, such as methanol, exceptionally efficient disproportionation of H_2_O_2_ is observed, due to the direct reaction of [(N_4_Py)Fe(iii)(OOH)]^2+^ with H_2_O_2_.^17,39^ Hence, in this case, although [(N_4_Py)Fe(iv)=O]^2+^ reacts with H_2_O_2_, it is not actually formed in the reaction and instead it is [(N_4_Py)Fe(iii)(OOH)]^2+^ that is responsible for the disproportionation of H_2_O_2_. In this example, the species responsible for substrate oxidation ([(N_4_Py)Fe(iv)=O]^2+^) is clearly different to that responsible for H_2_O_2_ disproportionation ([(N_4_Py)Fe(iii)(OOH)]^2+^). However, it is an interesting example to show how reaction modelling is important in revealing details of the overall catalytic system.

**Fig. 7:**
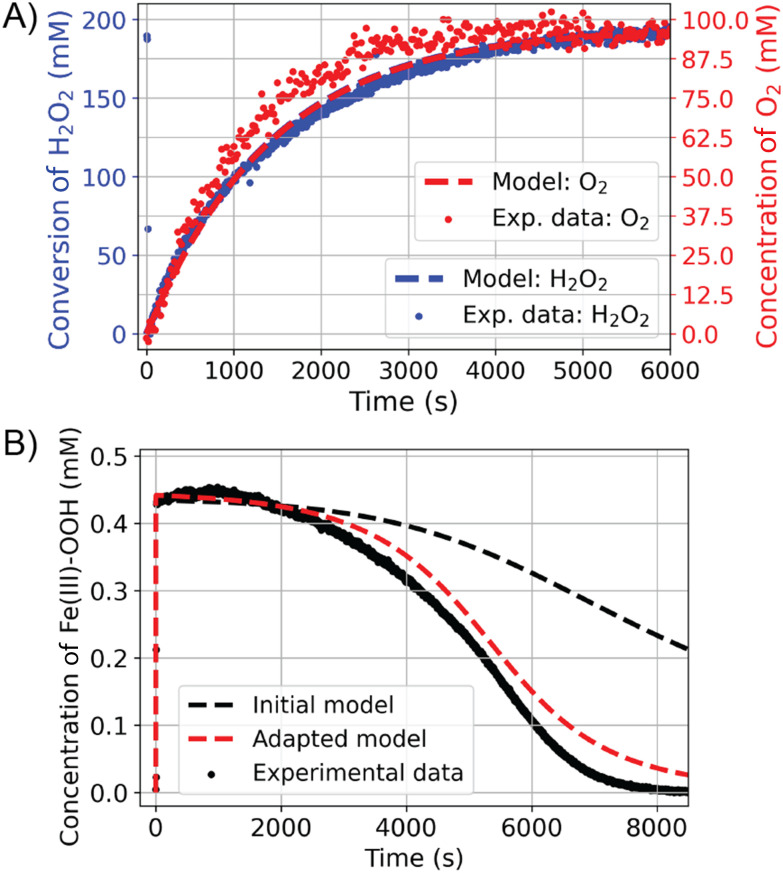
Reaction progress for the conversion of 400 equiv. of H_2_O_2_ (0.46 mM [(N_4_Py)Fe^II^ (CH_3_CN)](OTf)_2_ and 190 mM H_2_O_2_ in methanol). (A) ^3^O_2_ (red) and H_2_O_2_ (blue) reaction progress determined by Raman spectroscopy. (B) Concentration of (N_4_Py)Fe^III^–OOH over time from visible absorption spectroscopy. Experimental data are denoted with dots and the modelled data with dashed lines.^17^

The reactivity of [(N_4_Py)Fe(ii)(CH_3_CN)]^2+^ with H_2_O_2_ was elucidated using a range of *in situ* spectroscopic techniques. Time-resolved (resonance) Raman spectroscopy, headspace Raman spectroscopy, and UV/vis absorption spectroscopy showed that [(N_4_Py)Fe(ii)(CH_3_CN)]^2+^ disproportionates H_2_O_2_ into H_2_O and O_2_ in the presence of an excess of H_2_O_2_.^17,39^ Specifically, resonance Raman and UV/vis absorption spectroscopy were used to track the concentration of [(N_4_Py)Fe(iii)(OOH)]^2+^ in solution, and liquid phase and headspace Raman spectroscopy the concentrations of H_2_O_2_ and O_2_, respectively. The oxygen atom mass balance revealed that all H_2_O_2_ is disproportionated to H_2_O and O_2_ under these conditions. Fig. 7 shows the results of the combined, time-resolved liquid phase, headspace Raman and UV/vis absorption spectroscopic approach to follow the concentrations of H_2_O_2_, O_2_, and iron species, respectively.^17^

The disproportionation of H_2_O_2_ is wasteful and can generate hazardous conditions in large-scale applications. Under some circumstances, disproportionation of H_2_O_2_ can involve the generation of the highly reactive species ^1^O_2_. Singlet oxygen can engage in Diels–Alder type cycloadditions with dienes, for example, but can also trigger radical chain reactions. In the case of disproportionation of H_2_O_2_ by [(N_4_Py)Fe(iii)(OOH)]^2+^, the generation of ^1^O_2_ during the reaction was checked for by concurrently monitoring the O_2_ released from the reaction by headspace Raman spectroscopy and by NIR luminescence spectroscopy to determine ^1^O_2_ concentrations in the liquid phase (*vide supra*).^17^ Quantification of the chemiluminescence and in particular determination of limits of detection are important and the MoO_4_^−^ catalyst, which produces ^1^O_2_ quantitatively, provides a reliable standard. In the case of [(N_4_Py)Fe(iii)(OOH)]^2+^, only ^3^O_2_ is generated.

**Fig. 8:**
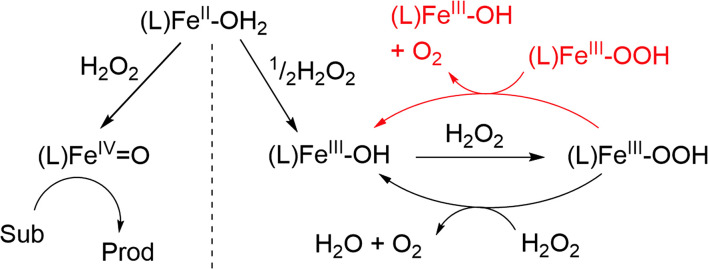
Adapted proposed mechanism for the reaction of [(N_4_Py)Fe^II^ (CH_3_CN)](OTf)_2_ with H_2_O_2_ in methanol, including an additional elementary step of the second order decay of (N_4_Py)Fe^III^–OOH (in red).^17^

The benefit of tacking concentrations of most if not all reaction components over time is that it allows for falsification/validation of proposed mechanisms. In this case, the proposed mechanism for H_2_O_2_ disproportionation was validated by comparing a microkinetic model constructed on the basis of the proposed mechanism with the experimental data (Fig. 7, dashed lines). In the kinetic model, the known individual rate constants and initial concentrations are used to predict the outcome of the reaction over time, with variation in any unknown rate constants, *etc*. to achieve a good fit to the experimental data. The outcome of the microkinetic modelling was consistent with the experimentally determined [H_2_O_2_] and [O_2_] over time (Fig. 7A). However, it did not agree with the time dependence of the concentration of [(N_4_Py)Fe(iii)(OOH)]^2+^ (Fig. 7B). This discrepancy prompted a closer look at the reactivity of [(N_4_Py)Fe(iii)(OOH)]^2+^ at lower concentrations, which revealed that in addition to the reaction with H_2_O_2_, the complex also reacts with itself to regenerate two equivalents of [(N_4_Py)Fe(iii)(OH)]^2+^ and an equivalent of H_2_O_2_. Furthermore the rate of homolytic O–O bond cleavage in [(N_4_Py)Fe(iii)(OOH)]^2+^ to form [(N_4_Py)Fe(iv)(O)]^2+^ and a hydroxyl radical was much lower than would be expected. Indeed this pathway is essentially irrelevant under normal reaction conditions as the self-decay of [(N_4_Py)Fe(iii)(OOH)]^2+^ to [(N_4_Py)Fe(iii)(OH)]^2+^ is faster than the formation of Fe(iv)=O from Fe(iii)–OOH (Fig. 8). Inclusion of the additional reaction step (the bimolecular reaction of [(N_4_Py)Fe(iii)(OOH)]^2+^) in the microkinetic model provided much closer agreement with all of the experimental data (Fig. 7).

### Switching from H_2_O_2_ disproportionation to organic oxidations through *in situ* changes in the catalyst structure

**Fig. 9:**
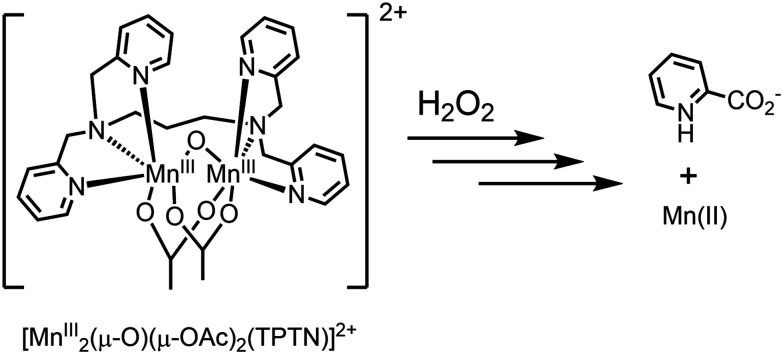
Decomposition of [Mn_2_(μ-O)(μ-OAc)_2_TPTN]^2+^ to pyridine-2-carboxylic acid.

A quite different situation arises where the initial form of a catalyst used is highly active in the disproportionation of H_2_O_2_ initially, but, following a change in the catalyst structure, the reactivity profile changes to the more desirable oxidation of organic substrates. We highlight this scenario with two manganese based catalysts applied already in the 2000s in the oxidation of organic compounds, namely [Mn_2_(μ-O)(μ-OAc)_2_TPTN]^2+^ (Fig. 9)^40,41^ and [Mn_2_(μ-O)_3_TMTACN_2_]^2+^ (Fig. 11).^42–47^

Both of these binuclear manganese complexes were inspired by nature's manganese dependent enzymes and the oxygen evolving centre of photosystem II, and each showed early promise in oxidation catalysis. However, both showed substantial inefficiencies due to the disproportionation of H_2_O_2_ during the oxidation of organic compounds, primarily alcohols and alkenes.

**Fig. 10:**
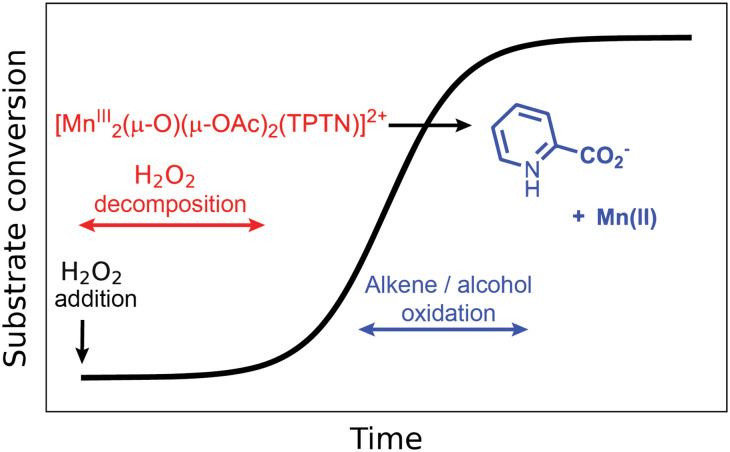
Reaction progress (conversion of the substrate) following addition of H_2_O_2_ to [Mn_2_(μ-O)(μ-OAc)_2_TPTN]^2+^.^40,41^

The oxidation of alkenes^40^ and alcohols^41^ with H_2_O_2_ in acetone catalysed by [Mn_2_(μ-O)(μ-OAc)_2_TPTN]^2+^ proceeds with good to excellent conversion, but required typically an 8-fold excess of H_2_O_2_ with respect to the substrate, due to excessive disproportionation to H_2_O and O_2_. It was noted also that a considerable lag period before oxidation of the substrate (*e.g.*, alcohol) begins. It is during the lag period that most of the H_2_O_2_ is lost due to comproportionation (Fig. 10).

Subsequent studies confirmed that during this lag period, the ligand underwent oxidative decomposition to pyridine-2-carboxylic acid (PCA). It was the PCA, together with manganese ions, that formed the catalyst that was responsible for the oxidation of organic compounds (*vide supra*), while the vigorous decomposition of H_2_O_2_ was due to the initial complex [Mn_2_(μ-O)(μ-OAc)_2_TPTN]^2+^.^48,49^

**Fig. 11:**
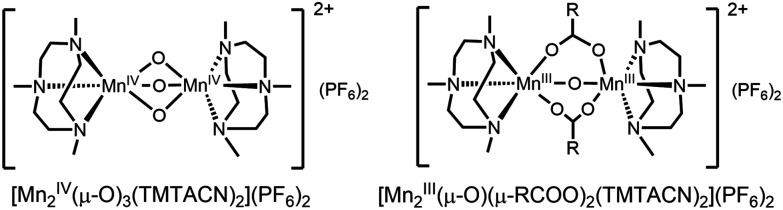
(Left) The Mn(iv) complex [Mn_2_(μ-O)_3_TMTACN_2_]^2+^ and (right) the Mn(iii) complex formed after a lag period following addition of H_2_O_2_ in the presence of a carboxylic acid.

**Fig. 12:**
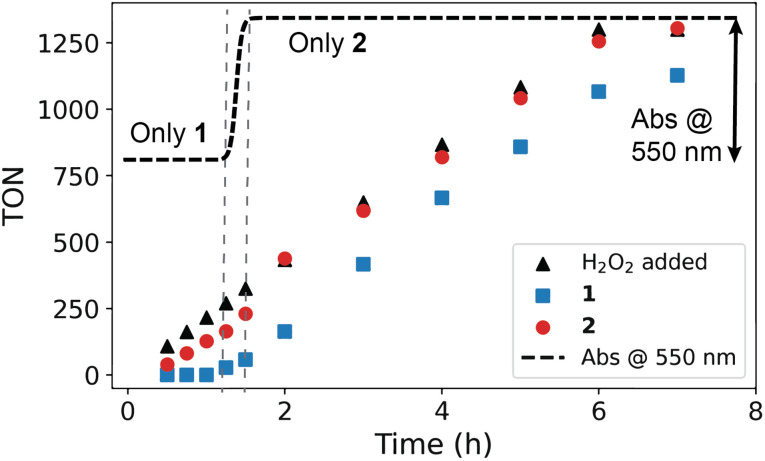
Reaction progress in the epoxidation of cyclooctene by dropwise addition of H_2_O_2_ with [Mn_2_(μ-O)_3_TMTACN_2_]^2+^ with 10 eq. of CCl_3_CO_2_H (1, blue rectangles) or [Mn_2_(μ-O)(μ-Cl_3_CO_2_)_2_(TMTACN)_2_]^2+^ (2, red circles). Adapted from de Boer *et al.*^46^ The turn-over-number (TON) for oxidation of substrate is plotted against the number of equivalents of H_2_O_2_ added (at a constant rate over time). The stepwise addition of H_2_O_2_ is indicated with a dashed black line. The complexes have different molar absorptivities at 532 nm and hence the conversion from one to the other can be followed over time by the absorbance at 532 nm (dashed line). The change in absorbance with 1 correlates with the switch to fully productive (alkene oxidation) use of H_2_O_2_.

**Fig. 13:**
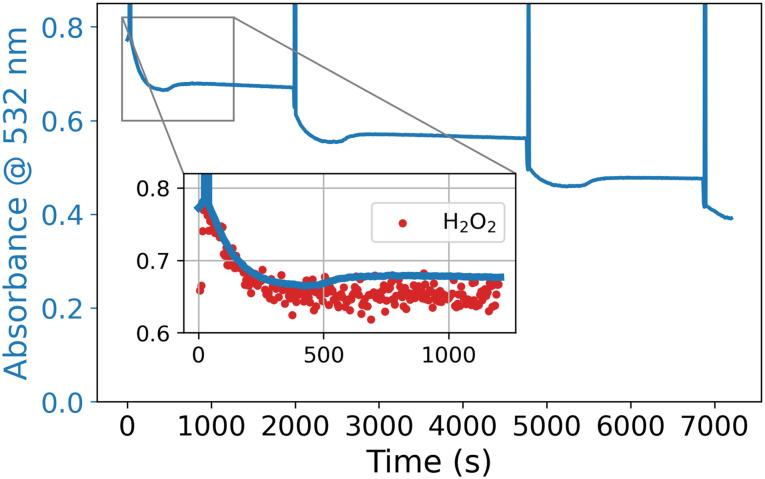
Absorbance at 532 nm over time with repeated addition of 50 equiv. of H_2_O_2_ to [Mn_2_(μ-O)(μ-RCO_2_)_2_(TMTACN)_2_]^2+^ (1 mM) with CCl_3_CO_2_H (10 mM) in acetonitrile (the spikes in absorbance denote the points at which H_2_O_2_ was added). Inset: overlay of absorbance at 532 nm (blue) and intensity of the Raman band of H_2_O_2_ (*λ*_exc_ 473 nm, red) at *ν̃* = 869 cm^−1^.

In contrast to [Mn_2_(μ-O)(μ-OAc)_2_TPTN]^2+^, the catalyst [Mn_2_(μ-O)(μ-OAc)_3_TMTACN_2_]^2+^ shows neither oxidation of organic substrates nor disproportionation of H_2_O_2_. In CH_3_CN containing carboxylic acids, however, a reproducible carboxylic acid dependent lag period is observed prior to a sudden and rapid conversion of [Mn_2_(μ-O)_3_ TMTACN_2_]^2+^ to [Mn_2_(μ-O)(μ-RCO_2_)_2_(TMTACN)_2_]^2+^ (Fig. 11, where R is an alkyl or aryl group).^45^ It is notable that with [Mn_2_(μ-O)(μ-RCO_2_)_2_(TMTACN)_2_]^2+^ (prepared independently), >95% efficiency in use of H_2_O_2_ for the epoxidation/*syn*-dihydroxylation of alkenes is observed (Fig. 12).^46^ However, in the short period where [Mn_2_(μ-O)_3_ TMTACN_2_]^2+^ converts to [Mn_2_(μ-O)(μ-RCO_2_)_2_(TMTACN)_2_]^2+^, in line monitoring the concentration of H_2_O_2_ revealed that disproportionation of H_2_O_2_ is significant, indicating that an intermediate complex is responsible (Fig. 13).^47^

Hence, this system is similar to the earlier TPTN/PCA based system in that the decomposition of H_2_O_2_ was primarily due to precursors to the final form of the catalyst that engages in the oxidation of organic compounds. However, in the absence of an oxidisable compound, the complex [Mn_2_(μ-O)(μ-RCO_2_)_2_(TMTACN)_2_]^2+^ engages in disproportionation of H_2_O_2_. This is seen by concomitant monitoring of the concentration of H_2_O_2_ by Raman spectroscopy and the catalyst by UV/vis absorption spectroscopy. In the absence of an organic substrate, the absorbance decreases as the H_2_O_2_ is disproportionated and only recovers partly indicating conversion to a Mn(ii) state followed by decomposition of the catalyst. This loss is not as pronounced when an alkene substrate is present.

## Conclusions

Disproportionation of H_2_O_2_ to O_2_ and H_2_O is a challenge in developing industrially applicable catalysts for the selective oxidation of organic substrates. Although it can be seen as a side reaction, typically ascribed to catalyst degradation, the study of several Mn- and Fe-catalysed oxidations that use H_2_O_2_ as a terminal oxidant has led to the realisation that H_2_O_2_ oxidation can occur *via* any of the several pathways. For example, H_2_O_2_ decomposition may occur *via* a reaction with the ‘activated catalyst’ (*i.e.*, that is directly responsible for the oxidation of the organic substrate), a resting catalyst state, catalyst degradation products, catalyst precursors, *etc*. Ultimately, it must be recognised that H_2_O_2_ is always likely to be a competitive substrate for oxidation.

Quantitative analysis of all reaction components, *i.e.*, catalysts, reagents and reaction products, is the ideal situation, allowing for full mass balance to be established, or at least that gaps in our knowledge of the fate of specific components are well established. Time-resolved spectroscopy techniques are especially useful to relate changes in selectivity to changes in catalyst composition and structure over the entire course of the reaction. This approach has allowed gaining insight into the many reasons why H_2_O_2_ decomposition can occur and keeping low steady state concentrations of H_2_O_2_ during catalysis may be of most importance in this regard. In the area of homogeneous oxidation catalysis with H_2_O_2_ as a terminal oxidant, studies aimed at tuning catalyst reactivity by ligand design, with respect to the organic substrate of interest, can be strengthened by taking into account the role of H_2_O_2_ disproportionation. In this aspect, there is an opportunity for computational studies to contribute in predicting selectivity.

## Author contributions

All authors contributed to the concept and writing of the manuscript.

## Conflicts of interest

There are no conflicts to declare.

## Data Availability

There are no original data associated with this manuscript.
